# Supplementary material for: Measuring Walking Speed Failed to Predict Early Death and Toxicity in Elderly Patients with Metastatic Non-Small-Cell Lung Cancer (NSCLC) Selected for Undergoing First-Line Systemic Treatment: An Observational Exploratory Study
Source: Cancers (Basel). 2022 Mar 5;14(5):1344. doi: 10.3390/cancers14051344 (PMC8909325; doi:10.3390/cancers14051344)
Supplement: Supplementary file 1 [file cancers-14-01344-s001.zip › cancers-1618329-supplementary.pdf]

Supplementary Table S1 Baseline characteristics of participants treated by chemo or immunotherapy with or without geriatric assessment

Characteristics	Geriatric Assessment		
	No<br>N = 19	Yes<br>N = 38	p-value
**Age, median (IQR)**	75 (73-78)	75 (72-79)	0.677
**Male sex, n (%)**	15 (79)	32 (84)	0.622
**Performance status**			
**0-1**	8 (42)	22 (58)	0.260
**2**	11 (58)	16 (42)	
**Smokers, n (%)**	16 (84)	34 (90)	0.360
**Pack years (IQR)**	48 (23-60)	50 (20-80)	0.325
**Histology**			
**Squamous, n (%)**	4 (21)	9 (24)	0.823
**Non-squamous, n (%)**	15 (79)	29 (76)	
**Weight, Kg (IQR)**	71 (68-78)	75 (65.8-85)	0.678
**BMI, Kg/m^2 (IQR)**	24.4 (22.5-29.5)	26.6 (23.5-29.5)	0.450
**Plasma albumin, g/L (IQR)**	36 (31-38)	39 (33-43)	0.064

Quantitative variables were expressed using the median, the 1rst and 3rd quartile (interquartile range= IQR), and categorical variables were given using the number and percentage, n (%). Quantitative variables were compared using the Wilcoxon test and categorical variables using the Chi-square or Fisher tests.
